# Characterization and verification of lead thickness of commercially available lead foil tape for the measurements of lead equivalency of radio‐protective shields

**DOI:** 10.1002/acm2.12891

**Published:** 2020-06-18

**Authors:** Pei‐Jan Paul Lin, Areej Fawzi Aljabal, Richard Ryan Wargo

**Affiliations:** ^1^ Division of Diagnostic Medical Physics Department of Radiology Virginia Commonwealth University Richmond VA USA; ^2^ Doctoral Program in Medical Physics Department of Radiation Oncology Virginia Commonwealth University Richmond VA USA

**Keywords:** lead thickness equivalence, lead and radio protective apparatus, transmission curves of lead

## Abstract

**Purpose:**

Radiation protective apparatus is normally specified in "millimeter" of lead equivalence. Typically, it is less than 0.5 mmPb with the exception of lead eyeglasses, which may be 0.75 mmPb equivalent. Upon discovery of commercially available lead foil tape, manufactured by 3M™ "Lead Foil Tape 421" (LFT) which is designed for industrial utility applications. We set out to determine if this LFT can, indeed, be employed as the reference lead in the evaluation of lead equivalency of various protective apparatus.

**Method:**

The LFT is cut to appropriate size (50 mm × 50 mm) and stacked for varying the total lead thickness for the transmission measurements. The transmission curves are obtained following the geometry spelled out in ASTM Designation F3094‐14 standards. The radiation beam qualities corresponding to modern cardiovascular angiography equipment in the range of 60~120 kVp, in increments of 10 kVp, and in combination with the spectral shaping filters of 0, 0.1, 0.2, 0.3, 0.6 and 0.9 mmCu were employed for characterization of the lead foil tape. The transmission data of lead pieces with known thicknesses (1/64", 1/32" and 3/64") are superimposed on the lead foil tape transmission curves to validate that the 3M™ LFT is indeed usable as 0.1 mm lead.

**Results:**

The transmission ratio (data points) of lead pieces with known thicknesses at various radiation beam qualities mentioned above, fall right onto the transmission curves of 3M™ LFT with better than 2% accuracy. Therefore, it is indeed behaving like 0.1 mm thick lead sheet, based on the superimposed transmission curves. The 3M™ "Lead Foil Tape 421" is employed as the reference lead for evaluation of radiation protective apparatus at this institution. Verification of lead protective apparatus with unknown lead equivalence can now be determined with a high accuracy and certainty.

## 1 Introduction

Lead and non‐lead radio‐protective garments and apparatus, hereafter radio‐protective shields (RPS), are widely employed for radiation protection of occupationally exposed personnel, and are found in various clinical environments where X‐ray imaging devices are utilized. Often, the lead equivalency (in millimeter of lead “mmPb”) of specific garment or apparatus cannot be identified due to the missing label, or the personnel needs to know if a given garment or apparatus comply with the minimum required lead thickness of 0.25 mmPb.^1^ For example, Code of Federal Regulations Title 21, Part 1000, Section 1000.50 (4) states that specific area gonad shielding should provide attenuation of X‐rays at least equivalent to that afforded by 0.25 mm of lead. However, the regulation of X‐ray‐producing devices is carried out by the individual states. Most states address the requirement for the personal protective equipment and may be slightly different from state to state.

In addition, specification of RPS is often rated with the beam quality at 80 kVp and 3.0 mm aluminum (mmAl) half‐value layer (HVL). The lead equivalency of these RPS at beam qualities other than 80 kVp/3.0 mmAl HVL is not well publicized, especially in the environment where cardiovascular interventional angiography (CIA) equipment are employed, where the spectral shaping filters are extensively utilized.^2^, ^3^ It is of great interest to determine what is the lead equivalency of these RPS in the real clinical environment. This study is attempting to evaluate thin (0.1 mm) lead foils that are essential for the transmission measurements.

In conducting the lead equivalency attenuation measurements, one basic problem would be encountered. In general, the thinnest commercially available lead sheet is 1/64” (0.3968 mm) with 1/32” and 1/16” lead backed gypsum boards as the most widely used for shielding of X‐ray rooms in radiology. But, the lead sheets (foils) needed to plot the transmission ratio should be approximately 0.1 mm in thickness so that the lead equivalency can be determined with high accuracy. However, lead sheets thinner than 1/64” are not readily available on the commercial market.

We have searched for lead foils for some time in the past few years. Thin lead foils may be purchased from scientific material supply companies such as GoodFellow^4^ and Alfa‐Aesar.^5^ However, even for the small quantity of lead foil required for this experimental study, it is relatively expensive. On the other hand, we have found that the lead foil tape manufactured by 3M™ might be a good substitute for lead foil that is 0.1 mm in thickness. The lead foil tape (LFT), product name: Lead Foil Tape 421, is designed for industrial utility applications^6^ and is readily available. According to the specification data sheets of 3M™ Lead Foil Tape 421, says the total thickness is 6.3 mils (0.16 mm) where the backing material (the lead foil) is 4 mils (0.1016 mm) thick and the rest is a thin layer of adhesive. This data sheet prompted us to determine if this industrial “adhesive tape”, 75 mm wide, would be suitable for transmission measurements as 0.1 mm lead.

## 2 MATERIAL AND METHODS

The 3M™ “LFT 421” is an adhesive tape. Proper handling of the LFT as a filter (foil) requires that the adhesives be either removed or covered up. Otherwise, when stacked together in layers, it would be difficult, if possible at all, to separate them while retaining their physical integrity. Instead of chemically removing the adhesives (2.3 mils, 0.00762 mm), we chose to adhere a thin layer of transparency film used for overhead projectors. Since both the coatings of adhesives on the LFT and the transparency film are thin, the effect of the attenuation rendered by both materials should be minimal, or may be ignored all together.

It should be noted that the radiation detection system employed is a Keithley pan‐cake type ionization chamber model 96030 (with an active volume of 15 cc) connected to an RTI chamber adapter and RTI Piranha electrometer. All are calibrated by the ANSI National Accreditation Board accredited calibration laboratory. The X‐ray imaging equipment employed is a CIA system (Siemens Artis Zee Bi‐plane system). It should also be pointed out that the tube potential accuracy of the CIA system was verified (better than 2% accuracy of selected value) as part of the annual inspection and prior to the execution of this study.

**Fig. 1:**
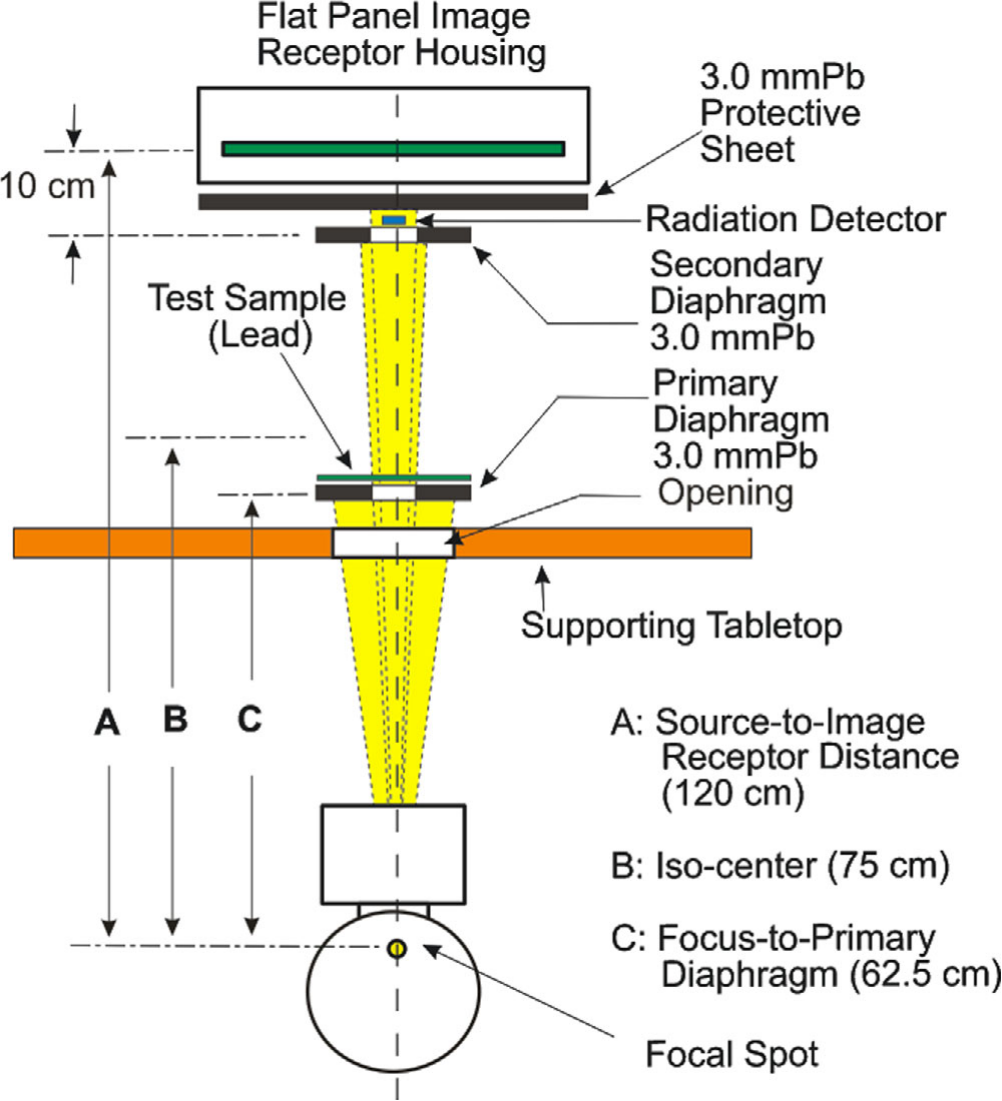
The narrow beam geometry employed for the transmission measurements. In order to take advantage of the clinically employed fluoroscopy system, the measurement geometry is modified to meet various mechanical constraints. Note that the supporting table is a homemade table with an opening in the tabletop designed for routine “in‐air” measurements of radiation output for compliance testing of regulatory requirements

In the preparation prior to the transmission measurements, the LFT is cut to an appropriate size (50 mm × 50 mm) and stacked together for varying the total lead thickness of up to 1.2 mm. The transmission curves are obtained by adapting the geometry set forth in American Society for Testing and Materials (ASTM) Designation F3094‐14,^7^ with minor modifications for the measurements. Modification was necessary as the radiation source employed for our experiment was an interventional angiography equipment installed for clinical applications. Depicted in Fig. 1 is the geometry utilized in the transmission measurements.

In order to verify whether this LFT is a valid substitute for 0.1 mm lead foil, the radiation beam quality encountered in the cardiovascular angiography procedures was replicated in the transmission experiment. A cardiovascular interventional angiographic equipment in clinical use was selected for the verification experiment. The angiographic system is equipped with dynamic spectral shaping filter mechanism driven by the automatic brightness control (ABC) or automatic dose rate and image quality (ADRIQ) control logic.^2^, ^3^, ^8^

It was necessary, however, to disable the ADRIQ control logic by entering into the “service mode”. Under the service mode, the exposure technique parameters can be manually adjusted for transmission measurements; (a) tube potential in the range of 60–120 kVp, in increments of 10 kVp, and in combination with (b) the spectral shaping filters of 0.0, 0.1, 0.2, 0.3, 0.6, and 0.9 mm copper (millimeter of copper; mmCu).

The transmission ratios of lead pieces with known thicknesses (1/64”, 1/32”, and 3/64”) are superimposed onto the transmission curves of 3M™ LFT 421 for validation. Since there are seven different tube potentials and five different copper filters, resulting in 35 combinations of differing beam qualities, characterization of the LFT 421 can be established if these three known thicknesses of lead sheets exhibit the same transmission ratios for the beam qualities in question.

## 3 Results

**Fig. 2:**
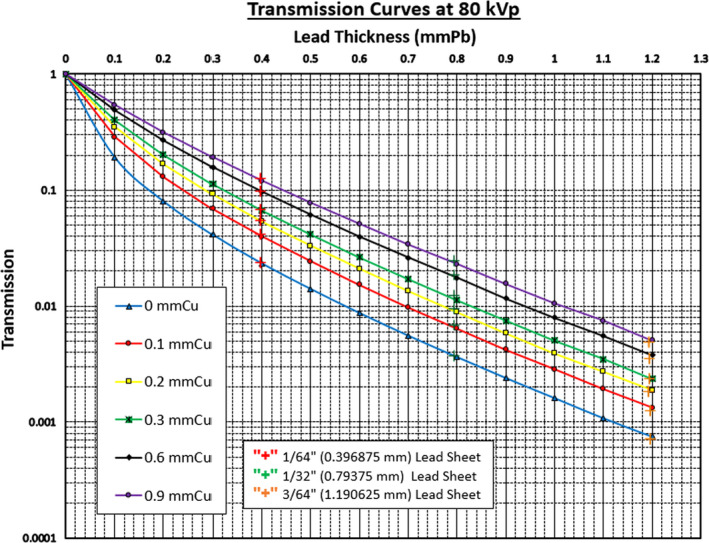
The transmission curves at 80 kVp for the spectral shaping filters. The transmission curves are for the 0.1 mm LFTs while the “+” markers are for the known lead thickness pieces under the same experimental conditions

**Fig. 3:**
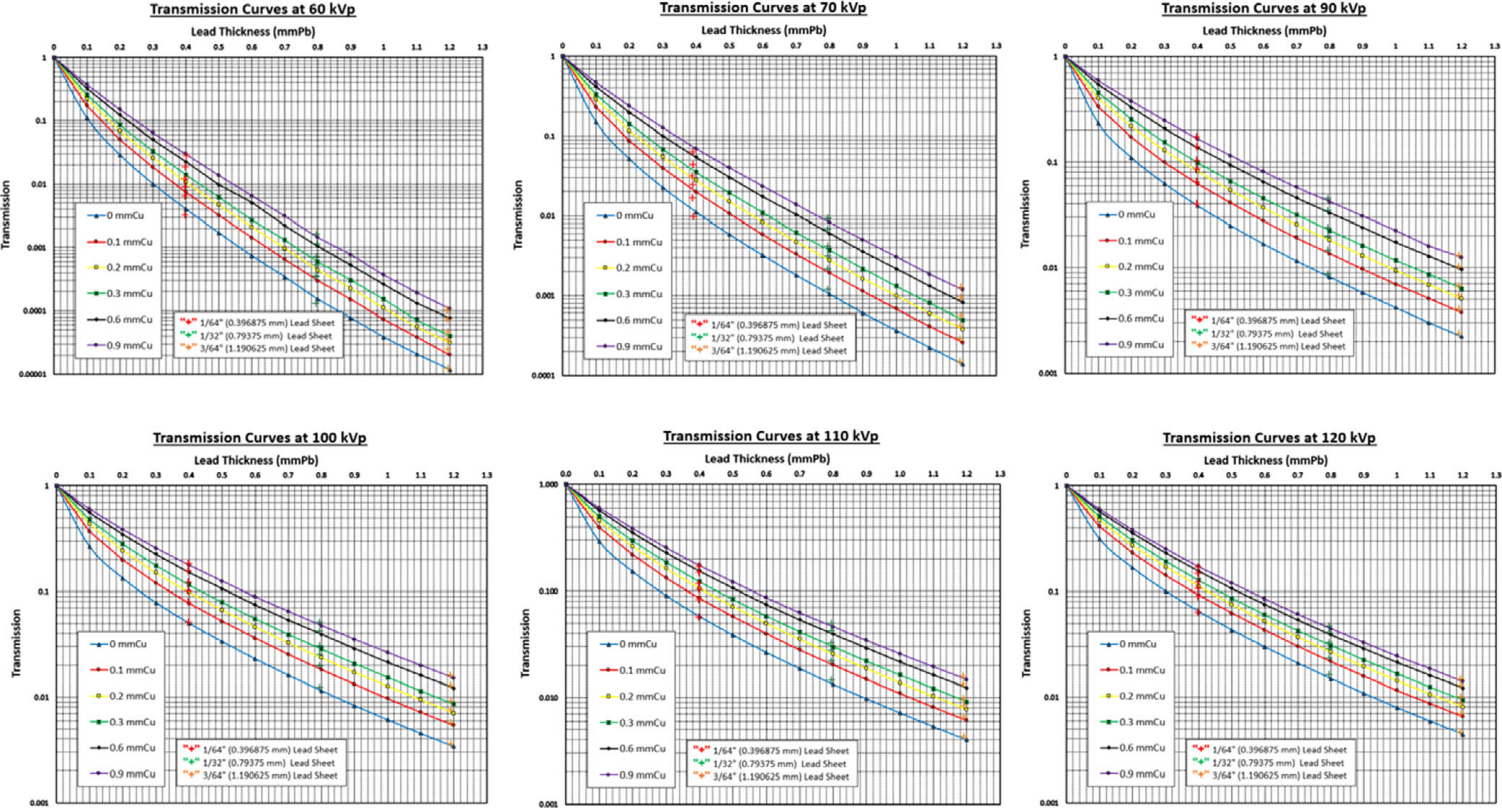
The transmission curves at various tube potentials. With these transmission curves, it is shown that the 3M™ LFT 421 is no different from the commercially available lead sheets employed in radiation protection shielding design

Depicted in Fig. 2, the transmission ratios of three lead pieces, shown as the “+” markers in the graphs, for the transmission curves of LFT 421 at 80 kVp. It is clearly shown that these 0.1 mm LFT pieces behave in the same manner as commercially available lead sheets. This very same characterization of LFT is further expanded to the conventional diagnostic radiology energy range. The measurement results are shown in Fig. 3 for all tube potentials from 60 kVp to 120 kVp (80 kVp is shown in Fig. 2).

Thus, the 3M™ Lead Foil Tape 421, despite the adhesives and the transparent overhead projection film, is indeed a viable alternative to plain 0.1 mm lead foils. It is obvious from these graphs that (a) the transmission ratio increases as the tube potential is increased, and (b) with respect to the spectral shaping copper filter, the thicker the Cu the higher the transmission ratio is.

## 4 Discussion

For the transmission measurements of LFT above a total thickness of 1.0 mmPb and with the 3/64” lead piece, the SID was reduced from 120 cm to 90 cm to gain radiation flux. This modification was necessary at the time of transmission measurements, specifically, for 60 kVp tube potential with 0.6 and 0.9 mm thick spectral shaping copper filters. This is due to the fact that the radiation detector employed in our investigation is not sufficiently sensitive to register any radiation under the measurement geometry.

We estimated the error bars both in the lead thickness and the transmission ratios. For the lead thickness, the manufacturing tolerances should apply which is ±0.005” (0.127 mm) irrespective of the nominal thickness. Thus, in the case of 1/64” lead sheet, the tolerances is approximately 30% of the normal thickness. Actual measurements using a micrometer showed a better than ±15% variation for the specific pieces employed for the measurement experiment. The specifications of lead pieces meet the Federal Specifications ASTM B749‐14^9^ and the IEC 61331‐1, Edition 2.0 (2014‐05) standards.^10^ The nominal lead thickness was employed when superimposing (plotting) the transmission ratios onto the transmission curves.

The micrometer measurements were performed with 8 points, 2 on each side, on the edges of the 50 mm × 50 mm square lead pieces, which might not represent the thickness in the middle of the square. However, it would be reasonable to assume that entire foil is uniformly in thickness within 10% of specified value (0.1 ± 0.01 mm). The error bar would be about the size of markers plotted on the graphs. The thickness of 3M^TM^ LFT is 0.1 mm with no tolerances listed in the product’s specification sheet. The transmission ratio error bar is better than 10% including the dosimeter reading reproducibility. Thus, the “+” marker on the graphs are in excess of ±10%.

## 5 Conclusions

The 3M^TM^ LFT is shown and characterized as having the same attenuation property as 0.1 mm of lead and is being employed at this institution to verify the lead equivalence of unknown protective apparatus. The samples, as submitted by the vendors, are evaluated with the 0.1 mm lead foils to estimate their lead equivalences over the entire range of diagnostic radiology tube potential range (60–120 kVp). Furthermore, the method provides information of lead equivalency regardless of the material used and that the data were provided for expected transmission for different X‐ray spectral configurations, that is, the beam quality under the spectral shaping filters of copper and other materials.

## Conflict of interests

There is no conflict of interest to declare.

## References

[1] Code of federal regulations title 21, Part 1000, Section 1000.50 (4). https://www.accessdata.fda.gov/scripts/cdrh/cfdocs/cfcfr/CFRSearch.cfm?fr=1000.50.

[2] Lin P‐JP . The operation logic of automatic dose control of fluoroscopy system in conjunction with spectral filters. Med Phys. 2007;34:3169–3172. 17879779 10.1118/1.2752576

[3] Lin P‐JP , Rauch P , Balter S , Fukuda A , Goode A , Hartwell G , Lafrance T , Nickoloff E , Shepard J , Strauss K . Functionality and operation of fluoroscopic automatic brightness control/automatic dose rate control logic in modern cardiovascular and interventional angiography systems. AAPM Report No. 125, 2012. 10.1118/1.4704524 22559654

[4] GoodFellow . http://www.goodfellow.com/A/Lead‐Foil.html/

[5] Alfa‐Aesar . https://www.alfa.com/en/catalog/012051/

[6] 3M™ Lead Foil Tape 421 and NPE‐IATD1095. https://www.3m.com/3M/en_US/company‐us/all‐3m‐products/~/3M‐Lead‐Foil‐Tape‐421/?N=5002385+3293242557&rt=rud

[7] ASTM Designation F3094–14 . Standard test method for determining protection provided by X‐ray shielding garments used in medical x‐ray fluoroscopy from sources of scattered X‐rays, American Society for Testing and Materials International, 100 Barr Harbor Drive.

[8] United States Patent 5,680,435 . X‐ray diagnostic apparatus with a filter device. Johann Seissl, October 21, 1997.

[9] ASTM Designation B749–14 . Standard specification for lead and lead alloy strip, sheet, and plate products, American Society for Testing and Materials International, 100 Barr Harbor Drive.

[10] IEC 61331–1, Edition 2.0 (2014–05) . Protective devices against diagnostic medical X‐radiation, Part 1: Determination of attenuation properties of materials. International Electrical Commission, 3 rue de varembe, P.O Box 131, CH‐1211 Geneva 20, Switzerland.

